# Cosmeceutical Ingredients From Plant Waste: Bioactive Extracts From *Vitis vinifera* L. branches, *Pinus sylvestris* L. and *Abies alba* Mill. Barks

**DOI:** 10.1002/cbdv.71916

**Published:** 2026-11-14

**Authors:** Marta Faggian, Silvia‐Maria Stanic, Giulia Galizia, Sara Ferrari, Gabriele De Nadai, Stefano Francescato, Giovanni Baratto, Gökhan Zengin, Alessandra Semenzato, Alina Yerkassymova, Stefania Sut, Stefano Dall'Acqua

**Affiliations:** ^1^ Unired srl Padova Italy; ^2^ Unifarco spa Belluno Italy; ^3^ Department of Biology Science Faculty Selcuk University Konya Turkey; ^4^ Department of Pharmaceutical and Pharmacological Sciences University of Padova Padova Italy

**Keywords:** anti‐inflammatory, antioxidant, grape branches, pine bark, plant waste, white spruce bark, whitening

## Abstract

In this paper hydro‐propanediol extracts targeted on skin hyperpigmentation were prepared from *Vitis vinifera* L. branches, *Pinus sylvestris* L. and *Abies alba* Mill. barks, derived from viticulture and forest industries. The three extracts were evaluated for contents of phenolic acids and flavonoids as well as for their antioxidant (DPPH, ABTS, CUPRAC, FRAP, phosphomolybdenum and metal chelating activity) activity. A detailed fingerprint of the extracts was obtained using UPLC‐QTOF and LC‐DAD‐MS^n^. More abundant compounds were stilbenes and proanthocyanidins in grape branches extract, sesquilignans and flavonoids in spruce and pine extracts. The anti‐tyrosinase effect of each extract and their mixture (1:1:1) were tested, demonstrating dose‐related response. The extract mixture was tested on re‐constructed human epidermidis for its anti‐inflammatory activity, showing a significant reduction (‐24%) of melanogenesis‐involved cytokine IL‐1α release induced by SLS irritation. Although additional studies are necessary, these findings imply that grape branches extract, pine bark extract, spruce bark extract, and their mixture could serve as novel and effective ingredients for counteracting melanogenesis. Various mechanisms of actions were highlighted, such as the inhibition of tyrosinase and the reduction of oxidative and inflammatory stimuli both linked to skin disorders associated with hyperpigmentation.

## Graphical abstract

Natural waste namely Pine and Sabies bark and *Vitis* branches have been extracted with hydro‐propanediol. Test were performed to assess antioxidant and metal chelating properties. Detailed chemical characterization was performed. A mixture of the three extract (1:1:1) was prepared. Mechanisms of actions linked to skin disorders associated with hyperpigmentation were highlighted, in particular the inhibition of tyrosinase and the reduction of oxidative and inflammatory stimuli.

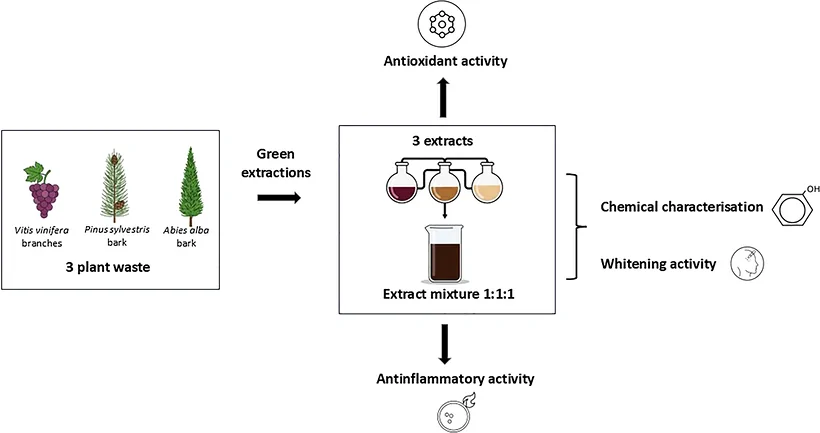

## 1 Introduction

Melanogenesis is a complex and highly regulated biological process responsible for the production of melanin, the pigment that imparts color to human skin, hair, and eyes [1]. This intricate pathway is not merely a cosmetic feature; it serves essential protective functions against ultraviolet (UV) radiation, which can lead to skin damage, oxidative stress, and an increased risk of skin cancers [2, 3, 4]. The molecular structure of melanin enables it to absorb a broad spectrum of UV and visible light, thus acting as a natural sunscreen that mitigates deoxyribonucleic acid (DNA) damage and reduces the risk of photocarcinogenesis [1, 2, 5]. Beyond its photoprotective role, melanin also plays a part in other physiological processes, including, antioxidant reactions, camouflage, and immune responses. The mechanisms governing melanogenesis are tightly controlled at multiple levels, involving genetic, enzymatic, and environmental influences, making it a critical area of study in dermatology, cosmetology, and medical research [2, 3, 6, 7].

Dysregulation of melanogenesis can lead to a variety of pigmentation disorders, broadly classified as hypo‐ and hyperpigmentation. Hypopigmentation disorders, such as vitiligo and albinism, result from defective melanin production or melanosome transfer, leading to patches of depigmented skin. Conversely, hyperpigmentation disorders, including freckles, solar and senile lentigines, and melasma, arise from excessive melanin deposition and uneven pigment distribution [8, 9]. These pigmentary conditions can significantly impact patients’ quality of life, contributing to psychological distress and social stigmatization [8, 10]. Consequently, both clinical and cosmetic fields prioritize the advancement of novel depigmenting agents and targeted treatments to more effectively and safely tackle pigmentation disorders.

In the cosmetic industry, natural plants‐derived extracts and compounds have been studied to identify active ingredients with a whitening action [11, 12, 13]. Melanin production can be influenced in different ways by phytoconstituents. The aromatic ring of plant‐derived phenolic compounds absorbs UVA and UVB radiation and consequently these compounds may be used as natural sunscreen agents to counteract the dangerous effects of UV exposure on human skin, which promotes melanogenesis [11]. Indeed, keratinocytes exposed to UV radiation are induced to produce proopiomelanocortin (POMC), which is the precursor of *alpha* and *beta* melanocyte‐stimulating hormones (*α*‐MSH, *β*‐MSH) and corticotropin, which stimulates the biosynthesis of melanin [12, 14]. Phytochemicals with melanin inhibitory effect by absorbing UV radiation are mostly flavonoids (quercetin, apigenin, luteolin derivatives), stilbenoids and resveratrol derivatives, caffeoylquinic acids, catechins and procyanidins [11].

In addition, biological signals regulated by nuclear factor erythroid 2‐related factor 2 (Nrf2) can affect the most relevant pathways involved in the regulation of photoaging and inflammation associated with skin barrier dysfunction and melanogenesis. Nrf2 activators upregulate genes encoding for antioxidant and protecting enzymes also involved in skin homeostasis, such as glutathione peroxidase, catalase, and superoxide dismutase. Various metabolites derived from plants such as the stilbenoids resveratrol and pterostilbene, sesquiterpene lactones (cynaropicrin), tannins, ginsenosides, isothiocyanate, fisetin but also carotenoids (lycopene, fucoxanthin) are reported to mediate a cytoprotective response in skin through the indirect antioxidant mechanism related to the promotion of Nrf2 activation and acting as anti‐photoaging and photoprotective agents [5].

Many phytochemicals have significant melanin inhibitory effects through the direct inhibition of tyrosinase enzyme, or the suppression of tyrosine oxidation mediated by tyrosinase interfering with melanin production. Such compounds belong to different chemical classes as the hydroquinone derivative arbutin, the flavonoids apigenin, nobiletin, luteolin, myricetin, kaempferol, quercetin, morin, iso‐rhamnetin, galangin, and their glycosides, the isoflavone daidzein and genistein, the chalcones as phloretin, the curcuminoids curcumin, desmethoxycurcumin, and the coumarins esculetin and stilbenes as resveratrol and derivatives [15]. Some phytochemicals are effective in melanogenesis inhibition through microphthalmia‐related transcription factor (MITF) downregulation and involving different signaling pathways, such as cyclic adenosine monophosphate/protein kinase A (cAMP/PKA), MSH by terpenes and ginsenosides, phosphatidylinositol 3 kinase/protein kinase B (PI3K/Akt) by curcumin and gallic acid, wingless‐related integration site (Wnt) /β‐catenin by flavonoids and mediating nitric oxide and melanosome autophagy by vanillic acid and β‐mangostine, respectively [11].

Plant secondary metabolites downregulate different inflammatory pathways and several mediators involved in melanogenesis. For example, quercetin inhibit IL‐18 in human keratinocytes [16]. Interleukin ‐18 (IL‐18) was associated with the promotion of melanogenesis through the protein kinase B/ nuclear factor kappa B (AKT/NF‐Κb) pathway, increasing tyrosinase activity and upregulating tyrosinase‐related protein 1 (TYRP‐1) and tyrosinase‐related protein 2 (TYRP‐2) expression [6]. Dihydroquercetin and some triterpenoids have been studied for their in vitro inhibition of matrix metalloproteinase ‐1 (MMP‐1) in UVB irradiation assay showing significant activities [17].

Curcumin, quercetin, kaempferol, and over 100 phytochemicals have been reported for the capacity to inhibit the production of prostaglandins such as Prostaglandin E2 (PGE‐2) many by reducing cyclooxygenase‐2 (COX‐2) gene expression, and others by acting as a COX‐2 inhibitors, blocking the stimulation of melanocyte dendrite formation through a cAMP dependent pathway [18].

In the search for new cosmeceutical agents targeted at skin hyperpigmentation, we considered three sources of phytochemicals as potential starting materials. In this regard, we selected plant sources containing compounds potentially useful to manage hyperpigmentation, with the idea to create new ingredients with a high level of sustainability. In fact, environmentally sustainable processes are becoming increasingly important in many industrial applications and are relevant in the cosmetic industry. Many cosmetic and cosmeceutical producers are developing strategies to counteract environmental impact, such as using sustainable raw materials and valorizing waste sources. To develop new natural active ingredients from waste natural materials, we selected three sources of phytochemicals: grape (*Vitis vinifera* L.) branches, Scots pine (*Pinus sylvestris* L.) bark, and white spruce (*Abies alba* Mill.) bark. Selection of the plant source was performed considering the composition of those barks that are rich in different phenolic constituents that have been considered as significant bioactive compounds as anti‐melanogenic agents [11, 12, 13].

*V. vinifera* L. (Vitaceae) is among the most widely cultivated fruit species in the world and originated from Asia and southern Europe [19]. Vineyard pruning leaves behind large amounts of woody branches each year, and this by‐product is a fairly rich source of stilbenoids [20]. Resveratrol and polydatin accumulate in grapevine as a stress response, and their levels shift with local agro‐climatic conditions [21]. Studies on grape cane chemistry point this plant part as a practical raw material for isolating trans‐resveratrol, alongside benzoic and hydroxycinnamic acids, catechin, epicatechin, and proanthocyanidins. Given both the traditional use of grapevine parts in Mediterranean folk remedies (for anemia, allergies, cold, and bronchitis) [22, 23] and this phytochemical profile, grape branches were a reasonable choice for the present analysis [19, 24]. A recent paper related to grapes described the effects of eleven different grape leaf extracts on tyrosinase and their sun protection factor [25]. Furthermore, grape waste from wine production has been recently studied for its possible application as a source of bioactive compounds for cosmetic use. Authors showed antioxidant activity, revealing radical scavenging capacity; antimicrobial activity, and inhibition of the activity of tyrosinase and elastase enzymes [26].

*P. sylvestris* L. (Pinaceae) is one of the most widely distributed conifers, and its bark has been chemically analysed in a fair number of studies [27]. The bark contains myricetin, eleutheroside, quercetin, vanillic acid, catechin, ferulic acid, and taxifolin, compounds that show antioxidant, fungicidal, anti‐inflammatory, and antibacterial activity [27, 28]. Combined with a long history of folk use for respiratory and skin complaints, this made Scots pine bark a natural candidate for inclusion in this study [29]. Extracts from the species *Pinus pinaster* have been shown to reduce in vitro melanin production by downregulating tyrosinase and reducing pigmentation‐related mediators, such as endothelin‐1 (ED‐1) and peroxisome proliferator‐activated receptor (PPAR) α, δ, and γ, therefore contributing to the inhibition of pathways associated with skin hyperpigmentation [30]. Pine species have recently been considered in a review indicating their biological activities and possible applications. Authors also underline the application of pine in food and the opportunity to implement the reuse of residual pine elements that, despite possible valuable applications, is still limited compared to its potential [31].

*A. alba* Mill. (Pinaceae) grows across the mountain ranges of Central and Southern Europe, and its bark has been studied in depth for its antioxidant chemistry [32]. Members of the genus *Abies* have been used as folk medicines against cold, stomach ache, indigestion, vascular, pulmonary, and venereal diseases [33]. That body of work, plus its traditional use for respiratory and rheumatic ailments, is why silver fir bark was included in this study. *A. alba* Mill has been considered in a recent review showing significant pharmacological activities, including antioxidant, antimicrobial, probiotic, antidiabetic, anti‐steatosis, and anti‐psoriatic activities, suggesting potential usefulness also in skin diseases [32]. White spruce has been considered together with other plants for the antioxidative, dermal immunomodulatory, and antimicrobial properties. The authors evaluated bark extracts from European common temperate trees considering various skin pathogens, wound healing, and the maintenance of skin health. Furthermore, there are plenty of uses in the ethnopharmacological tradition of several countries, showing the importance of this species as a source of possible bioactive compounds [34].

With the aim of developing innovative cosmeceutical whitening agents acting on the inhibition of the melanogenesis pathway involving oxidation, inflammation, and melanin production, we considered waste natural materials, namely the grape (*V. vinifera* L.) branches, Scots pine (*P. sylvestris* L.) bark, and white spruce (*A. alba* Mill.) bark. The selection of these species is based on the literature indicating these plants as significant sources of bioactive phenolics and the availability of the materials in the northern part of Italy to create a possible sustainable supply chain for the natural product. Extraction will be performed using water (30%) and 1,3‐propanediol (70%) as extraction vehicles. Phytochemical characterization and bioassays targeted on antioxidant activity and on the melanogenesis process have been performed.

## 2 Materials and Methods

### 2.1 Plant Materials and Production of Botanical Extracts

*V. vinifera* L. branches were provided by Il Brolo Società Agricola Impresa Sociale srl farm (Teolo, Padova, Italy). *P. sylvestris* L. bark was provided by Segheria Traiber sawmill, (Forno di Zoldo, Belluno, Italy). *A. alba* Mill. bark was provided by Zoppè Vania sawmill, (Alpago, Belluno, Italy). Identity of plant samples were assessed by taxonomist. A voucher sample of each material is stored at DSF University of Padova, *V. vinifera* L. branches (VVB24), *P. sylvestris* L. bark (PSB25), *A. alba* Mill. Bark (AB2024).

The vegetal material was dried at 38°C until reaching a humidity lower than 10% and then it was grinded using a mill with a 4 mm mesh sieve. The three different botanical species were processed separately. Grinded vegetal material was extracted for 1 h at room temperature using a solvent mixture composed of 1,3‐propanediol (DuPont Tate & Lyle Bio Products Company, Loudon, (TN), USA) and water (70% w/w, 30% w/w, respectively) with an ultrasonic extractor. The drug‐solvent ratio was 1:10 w/w.

Vegetal material was removed by filtration, and the extracts were clarified by progressive filtration.

The extract mixture was obtained by mixing grape branches extract, pine bark extract, and spruce bark extract in equal proportions (1:1:1).

### 2.2 Determination of Total Phenolic Content and Flavonoid Content

For the analysis of total phenolic contents, the adopted protocol was the following: the sample solution (3 mg/mL) was mixed with a solution of the Folin–Ciocalteu reagent (dilution 1:9) and shaken vigorously. After 3 min, 1 mL of a 1% solution of Na_2_CO_3_ was added. Absorbance was read at 760 nm after a 1‐h incubation at room temperature, protected from light. The total phenolic content was expressed as milligrams of gallic acid equivalents (mg GAE/g extract) [35].

To measure the total flavonoid content the sample solutions (3 mg/mL) were mixed with the same volume of AlCl_3_ solution at a concentration of 2% (w/w) in methanol. The blank solutions were prepared by adding sample solutions (1 mL) to methanol (1 mL) without AlCl_3_. After 10 min of incubation at room temperature the absorbances of samples and blanks were read at 415 nm. A reference curve was obtained using rutin solutions (10‐400 µg/mL). The total flavonoids content was expressed as equivalents of rutin (REs) [36].

### 2.3 Free Radical Scavenging Assays

For the 1,1‐diphenyl‐2‐picrylhydrazyl (DPPH) radical scavenging assay, the solution of the sample (5 mg/mL) was added to 4 mL of a methanol solution of DPPH (3 mg/100 mL). The sample absorbance was read at 517 nm after a 30 min incubation at room temperature, in the dark. Scavenging activity was expressed as milligrams of trolox equivalents (mg TE/g extract) [37].

For 2,2′‐azino‐bis(3‐ethylbenzothiazoline) 6‐sulfonic acid (ABTS) radical scavenging assay, ABTS+ was produced directly by reacting 7 mM ABTS solution with 2.45 mM potassium persulfate and allowing the mixture to stand for 15 (min) in the dark, at room temperature. Prior to beginning the assay, ABTS solution was diluted with methanol to an absorbance of 0.700 ± 0.02 at 734 nm. Sample solution was added to ABTS solution (2 mL) and mixed. The sample absorbance was read at 734 nm after a 30 min incubation at room temperature. The ABTS radical scavenging activity was expressed as milligrams of trolox equivalents (mg TE/g extract) [38].

### 2.4 Reducing Power Assays

The Phosphomolybdenum assay was performed using 5 mL of reagent solution (0.6 M sulfuric acid, 28 mM sodium phosphate and 4 mM ammonium molybdate) that was mixed with 1 mL of sample solution (0,3 mg/mL). The sample absorbance was read at 695 nm after a 90 min incubation at 95°C. The measurement was expressed as milligrams of trolox equivalents (mg TE/g extract) [39].

For the cupric ion reducing activity (CUPRAC) activity assay, the protocol applied was the following. Sample solution (1 mg/mL; 0.5 mL) was added to reaction mixture containing CuCl_2_ (1 mL, 10 mM), neocuproine (1 mL, 7.5 mM), and NH_4_Ac buffer (1 mL, 1 M, pH 7.0). A blank solution was prepared by adding sample solution (0.5 mL) to reaction mixture (3 mL) but in the absence of the CuCl_2_ solution. Solution is kept for 30 min at room temperature. Absorbances of the solutions are measured at 450 nm. CUPRAC activity was expressed as milligrams of Trolox equivalents (mg TE/g extract) [40].

The ferric reducing antioxidant power (FRAP) was measured mixing the FRAP reagent (acetate buffer 0.3 M, pH 3.6), 2,4,6‐tris(2‐pyridyl)‐S‐triazine (TPTZ) (10 mM, in 40 mM HCl) and ferric chloride (20 mM) in a ratio of 10:1:1 (v/v/v). After incubation at room temperature for 30 min the absorbance is measured at 593 nm. Trolox is used as reference and FRAP activity was expressed as milligrams of trolox equivalents (mg TE/g extract) [41].

### 2.5 Metal Chelating Assay

To establish the metal chelating ability, the sample solution (1 mg/mL; 2 mL) was added to FeCl_2_ solution (0.05 mL, 2 mM). Then 5 mM ferrozine (0.2 mL) was added. Blank solution was prepared by adding sample solution (2 mL) to FeCl_2_ solution (0.05 mL, 2 mM) and water (0.2 mL), without ferrozine. Then, the sample and blank absorbances were read at 562 nm after 10 min incubation at room temperature. The absorbance of the blank was subtracted from that of the sample. The metal chelating activity was expressed as milligrams disodium edetate (EDTA) equivalents (mg EDTAE/g extract) [42].

### 2.6 LC–DAD‐MS^n^ Analysis of Grape Branches, Pine and Spruce Bark Extracts

Liquid Chromatography diode array and multiple stage mass spectrometry (LC‐DAD‐MS^n^) analyses were obtained using a 1260 Agilent LC system (Santa Clara, MA, USA) equipped with DAD, autosampler, and column oven. After the chromatographic column, a “T” connection divided the flow equally to DAD and MS. As mass spectrometer, a Varian MS 500 Ion trap equipped with Electrospray Ion Source (ESI) was used, working both in negative and positive ion mode and acquiring the data in the *m/z* range 100–2,000. Fragmentation of most intense ion species was obtained using the turbo data‐dependent scanning (tdds) function of the instrument.

For the analysis of grape branches extract, pine bark extract, spruce bark extract and relative mixture (1:1:1), the samples were diluted in water (1:10). MS parameters were as follows: spray shield, 600 V; nebulizer pressure, 25 psi; drying gas pressure, 15 psi; capillary voltage, 80 V; RF loading, 80%; needle voltage, 4,500 V. An Agilent SB‐C18 column (4.6. × 50 mm, 3.5 µm) was used as stationary phase. Solvents were: 1% formic acid in water (A), acetonitrile (B) and methanol (C). Gradient was as follows: Minute 0, 95:5:0 (% A:B:C), Minute 2.5, 85:15:0 (% A:B:C), Minute 12, 80:18:2 (% A:B:C), Minute 15.5, 50:40:10 (% A:B:C), Minute 19, 20:70:10 (% A:B:C), Minute 21, 0:85:15 (% A:B:C), Minute 23, 0:85:15 (% A:B:C), Minute 24, 0:100:0 (% A:B:C), Minute 26, 0:100:0 (% A:B:C). The flow rate was 0.75 mL/Minute. As reference compounds for quantitative analysis, trans ‐ε‐viniferin, piceatannol, ω‐viniferin, viniferin (Sigma‐Aldrich, Milan, Italy) was employed for the quantification of stilbenoids in grape extract. Catechin, epicatechin, gallocatechin, epigallocatechin (Sigma‐Aldrich) procyanidin B2 (Extrasynthese, Genay Cedex, France) were employed for the quantification of monomeric and oligomeric flavan‐3‐ols in grape, spruce, and pine extracts. Taxifolin (Sigma‐Aldrich) was employed for the quantification of flavonoids in pine extract, lariciresinol and pinoresinol (Sigma‐Aldrich) were employed for the quantification of sesquilignans in pine and spruce extract. Gallic acid and ellagic acid (Sigma‐Aldrich) were employed for the quantification of gallic acid derivatives in spruce extract. For the terpene derivatives due to lack of specific reference available compound, abietic acid was used. For the semiquantitative analysis the calibration curves were as follows: Catechin (100‐5 µg/mL) y = 8.32e+2 x‐ 0.05, epicatechin (100‐5 µg/mL) y = 8.21e+2 x‐ 0.04, gallocatechin (120‐6 µg/mL) y = 7.65e+2 x‐ 0.10; epigallocatechin (110‐2.2 µg/mL) y = 6.12e+2 x‐ 0.11; procyanidin B2 (100‐1 µg/mL) y = 3.11e+2 x‐ 0.05, Taxifolin (120‐1,2 µg/mL) y = 4.2e+3‐0.02, Lariciresinol (50‐5 µg/mL) Y = 1.23e+2 +0.002 pinoresinol (80‐2 µg/mL) Y = 4.12e+2 +0.02, abietic acid (150‐3 µg/mL) Y = 1.23e+1+0.22. Identification of compounds was obtained comparing the MS fragmentation spectra with the literature, and MS and retention times (RT) with those of available standard compounds.

### 2.7 UPLC‐QTOF Analysis of Grape Branches, Pine and Spruce Bark Extracts

To confirm the identity of the compounds also Ultra Performance Liquid Chromatography coupled with Quadrupole‐Time of Flight Mass detector (UPLC‐QTOF) was used.

A Waters H‐Class UPLC was used as chromatograph and a XEVO QTOF as mass spectrometer. The stationary phase was a Waters BEH C18 2.1×150 mm 1.7 µm. The mobile phases were water 0.1% formic acid (A), Acetonitrile (B) and Methanol (C). Flow rate was 0.3 mL/min. Gradient was as follows: Minute 0, 95:5:0 (% A:B:C), Minute 2, 95:5:0 (% A:B:C), Minute 14.5, 10:15:75 (% A:B:C), Minute 15, 10:15:75 (% A:B:C), Minute 16.5, 0:25:75 (% A:B:C), Minute 22, 0:25:75 (% A:B:C), Minute 22.1, 95:5:0 (% A:B:C), Minute 24, 95:5:0 (% A:B:C).

Mass spectrometer was operating in negative ion mode using the MS^e^ function that allows the acquisition of fragmentation spectra. The ion source operated at 450°C of temperature of drying gas with pressure of 700 L/Hrs and 20 psi of collision gas. Accurate mass measurement was corrected with the use of lock spray solution (Leukin Encephalin) directly infused in the source at flow rate of 20 µL/Minute. Identification was obtained matching the accurate MS measurements and fragments with database or with reference compound.

### 2.8 Determination of Tyrosinase Inhibitory Effect in Grape Branches Extract, Pine Bark Extract, Spruce Bark Extract and Extract Mixture (1:1:1)

The inhibitory effects of grape branches extract, pine bark extract, spruce bark extract and relative extract mixture (1:1:1) on tyrosinase activity were determined as follows, according to previous paper with modification [36]: 2.8 mL of L‐Dopa solution (100 µg/mL in 50 mM Na_2_HPO_4_‐NaH_2_PO_4_ buffer, pH 6.8, Sigma‐Aldrich) and 0.1 mL of mushroom tyrosinase (EC 1.14.18.1, >1000 U/mg, Sigma‐Aldrich) solution (170 µg/mL in 50 mM Na2HPO4‐NaH2PO4 buffer, pH 6.8) were added to 0.1 mL of single extracts or extract mixture as is and diluted 2, 3 and 5 times in water, arriving to the final concentrations of 33.3, 16.7, 11.1 and 6.7 mg/mL. As reference standard, 0.1 mL of kojic acid solutions (1‐0.5‐0.2‐0.1 mg/mL in DMSO) were added to 2.8 mL of L‐Dopa and 0.1 mL of tyrosinase solutions, arriving to final concentrations of 33.3, 16.7, 6.7, 3.3 µg/mL. Finally, the mixture of L‐Dopa and tyrosinase solutions was employed as blank solution, by replacing tested samples with 0.1 mL of phosphate buffer. Enzyme inhibitory activity was determined by measuring the absorbance values at 475 nm, monitoring the oxidation of L‐DOPA to dopachrome. The reaction was carried out at a constant temperature of 30°C, for six min. The absorbance of blank solution refers to 100% tyrosinase activity. The percentage of tyrosinase inhibition was calculated as follows: 100 × [OD(475 nm) test sample] / [OD(475 nm) blank solution].

### 2.9 In Vitro Cell Viability and Evaluation of Soothing Efficacy on Reconstructed Human Epidermidis

The objective of this assay was to assess the effects of the mixture of grape branches, pine, and spruce bark extracts (1:1:1) in protecting skin cells and in inhibiting the inflammatory reaction caused by moderate irritating agents such as sodium lauryl sulfate (SLS) on a multilayer in vitro reconstructed human epidermis cell model. The inhibition of the SLS‐induced release of Interleukin‐1 alpha (IL‐1α) cytokine was investigated through specific enzyme‐linked immunosorbent assay (ELISA) following exposure to the extract mixture and compared to untreated epidermis.

A reconstructed artificial human skin model (SkinEthic RHE, Episkin, Lione, France) comprising normal human epidermal keratinocytes, growing as an integrated three‐dimensional (3D) cell culture model was employed. All bioassays were performed in triplicate, and results are expressed as average of the three independent tests.

Bioassays were initiated by evaluating cytotoxicity of various materials to assess the cell viability and the compatibility of the sample with the test (24 h exposure). 3‐[4,5‐dimethylthiazol‐2‐yl]‐2,5‐diphenyltetrazolium bromide (MTT) cytotoxicity assay was performed on phosphate buffer (PBS) as negative control, on SLS 0.4% as positive control, on 1,3‐propanediol (to rule out adverse effects due to its presence in the extraction vehicle), on extract mixture (1:1:1) alone and on extract mixture (1:1:1) + 0.4% SLS. Epidermis units are treated with 1 mg/mL MTT solution for 3 h at 37°C. The solution is then removed and replaced with isopropanol and the units are further incubated for 2 h at room temperature with medium shaking. 2 aliquots of every sample are transferred to a 96‐well plate for the reading. The optical density (OD) is read at the wavelength of 570 nm with a colorimeter (Tecan model Infinite F200) equipped with a microplate reader [43]. The percentage of mean cell viability was calculated as follows: [OD(570 nm) test sample / OD(570 nm) negative control] × 100.

For the IL‐1α assay, epidermis units were pre‐treated for 60 min with 0.4% SLS to induce IL‐1α synthesis and release. Tissues were then washed with PBS and then 40 µL of the mixture of the three undiluted extracts were applied. As negative control, epidermis units have been treated with PBS, while units treated with SLS only have been used as positive control. Units treated with the extract mixture only were used as further control. Test was carried out in triplicate. The exposure has been carried out for 24 h at 37°C, 5% carbon dioxide (CO_2_). At the end of the exposure period, samples were removed by PBS washing, and the culture medium has been collected for IL‐1α assay.

IL‐1α has been determined in the medium culture of treated and not treated epidermis, using a direct ELISA (Sigma Aldrich) test. The colorimetric signal is directly proportional to the amount of cytokine in the culture medium. Samples absorbance is read at 450 nm. The sensitivity limit is less than 10 pg/mL. Cytokine concentration is determined using a standard curve. % IL‐1α inhibition is determined as follows: 100‐ (pg/mL IL‐1 α release by irritated sample/ pg/mL IL‐1α released by positive control) × 100.

## 3 Statistical Evaluation

All the experiments were carried out in triplicate. Considering the phytochemical composition, data are expressed as average ± standard deviations (SD). Considering the in vitro assay the IC_50_ were expressed as average ± SD. One‐way ANOVA followed by Duncan's post hoc test for multiple comparisons with blank or control, was adopted to compare the differences. Statistical analyses were performed using SPSS 16.0 software. A value of p < 0.05 was considered to indicate statistical significance.

## 4 Results

### 4.1 Total Phenolics Content, Total Flavonoids Content, and Antioxidant Activity Assays in Grape Branches, Pine Bark, and Spruce Bark Extracts

The findings from the spectrophotometric analysis regarding total phenolics and flavonoids content as well as antioxidant activities and reducing properties of grape branches, pine bark, and spruce bark extracts are outlined in Table 1.

**TABLE 1:** Total phenolics and flavonoids content, antioxidant activities and reducing properties of grape branches, pine and spruce bark extracts (GAEs: Gallic acid equivalents, REs: Rutin equivalents, TEs: Trolox equivalents, EDTAEs: EDTA equivalents), different letters indicate statistical differences in the columns (p<0.05).

Assay	Grape branches extract	Pine bark extract	Spruce bark extract
Total phenolics content (mg GAEs/g)	7.07 ± 0.34^a^	19.13 ± 0.06^b^	7.16 ± 0.18^a^
Total flavonoids content (mg REs/g)	0.58 ± 0.09^a^	0.75 ± 0.06^b^	0.20 ± 0.04^c^
DPPH (mg TEs/g)	21.82 ± 0.34^a^	82.68 ± 1.04^b^	31.45 ± 0.96^c^
ABTS (mg TEs/g)	30.72 ± 1.04^a^	114.20 ± 3.42^b^	42.24 ± 1.76^c^
Phosphomolybdenum (mg TEs/g)	0.27 ± 0.02^a^	0.60 ± 0.06^b^	0.22 ± 0.02^a^
Metal chelating (mg EDTAs/g)	0.00	0.00	0.00
CUPRAC (mg TEs/g)	21.36 ± 0.33^a^	77.99 ± 0.28^b^	26.55 ± 1.13^c^
FRAP (mg TEs/g)	18.63 ± 0.47^a^	61.11 ± 1.38^b^	24.26 ± 2.59^c^

The total phenolics content varies between 7 mg GAEs/g in grape branches and spruce bark extracts to 19 mg GAEs/g in pine bark extract. The total flavonoid contents of the extracts range from 0.20 to 0.75 mg REs/g. Considering antioxidant efficacy and reducing power of three single extracts, the pine bark extract demonstrated the highest activities across all performed assays (DPPH, ABTS, Phosphomolybdenum, CUPRAC, FRAP assays), whereas the grape and spruce extracts showed activities that were three to four times lower than those of pine bark. Each extract also displayed no chelating activity against transition metals.

### 4.2 LC–DAD‐MS^n^ Analysis of Grape Branches Extract, Pine Bark Extract, Spruce Bark Extract and Extract Mixture (1:1:1)

The phytochemical composition of grape branches, pine, and spruce bark extracts was investigated with LC‐DAD‐MS^n^ approach and compounds were identified using UV absorption and mass spectrometry approaches, as well as injection of reference compounds, when available. The chromatogram of grape branches extract is reported in Figure 1 and the quali‐quantitative composition is reported in Table 2.

**FIGURE 1:**
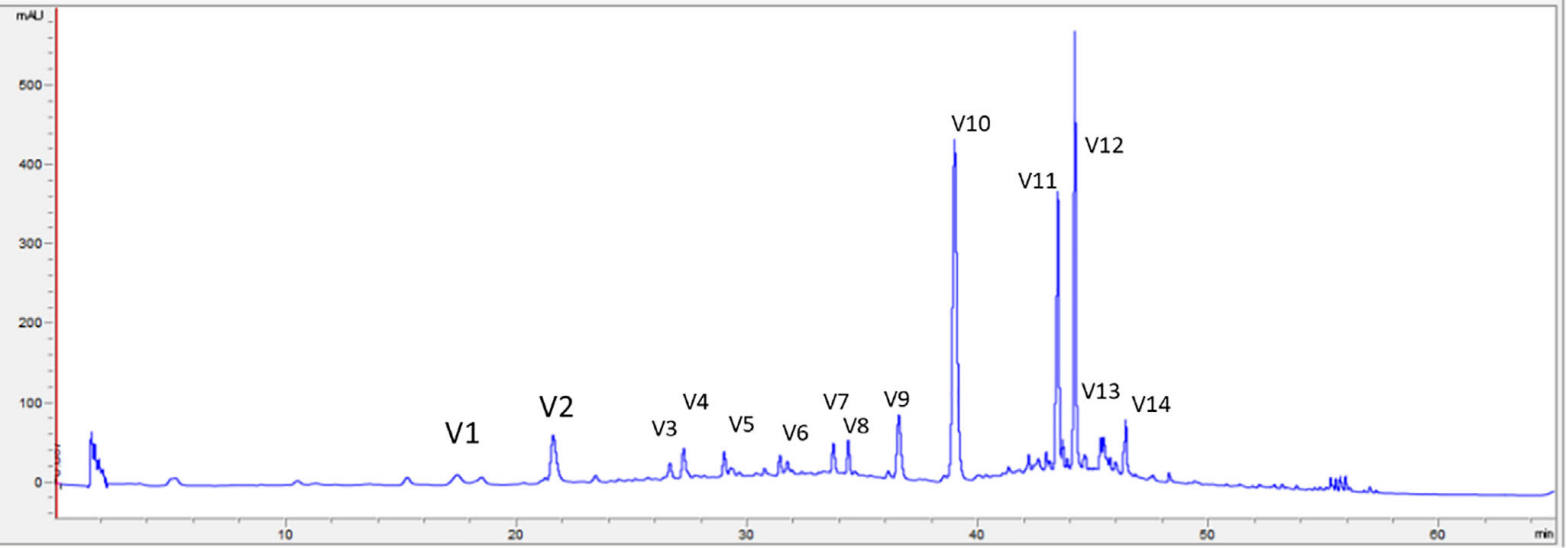
LC chromatogram of grape branches extract. Numbers are referred to the identified compounds as reported in Table 2.

**TABLE 2:** Monomeric and oligomeric flavan‐3‐ol derivatives (FD) and stilbenes (ST) detected in grape branches extract using LC‐DAD‐MS^n^ approach. Compounds indicated with * were confirmed by reference standard injection.

N.	RT [min] LC IT	HR [M‐H]^−^ UPLC‐QTOF and molecular formula	MS/MS fragments	Annotated or Identified compounds	Chemical class	µg/mL
V1	18.4	577.1343 C_30_H_46_O_12_	425, 407, 289, 451, 287, 245, 451	Procyanidin B dimer	FD	7.43 ± 0.25
V2	21.3	289.0713 C_15_H_14_O_4_	245,205, 203, 179, 205, 227, 109, 123, 165, 125, 151	Catechin*	FD	44.56 ± 1.48
V3	26.8	471.1443 C_28_H_24_O_7_	349, 375	Stilbenoid dimer	ST	1.56 ± 0.02
V4	27.1	289.0712 C_15_H_14_O_4_	245, 203, 179	Epicatechin*	FD	16.24 ± 0.28
V5	29.5	575.1193 C_30_H_24_O_12_	407, 289, 449, 285	Procyanidin A dimer	FD	24.04 ± 0.57
V6	31.2	469.1287 C_28_H_22_O_7_	451, 423, 375, 363, 357, 345, 335, 269 229, 330, 301	Ampelopsin A	ST	1.33 ± 0.02
V7	33.5	243.0675 C_14_H_12_O_4_	201, 159	Piceatannol*	ST	2.47 ± 0.01
V8	34.3	453.1340 C_28_H_22_O_6_		Viniferin*	ST	2.17 ± 0.01
V9	36.4	905.1996 C_56_H_42_O_12_	811, 717, 611, 357, 675, 568, 488, 359	Hopeaphenol	ST	6.94 ± 0.08
V10	38.8	905.1996 C_56_H_42_O_12_	811, 717, 611, 503, 357	Isohopeaphenol	ST	38.93 ± 1.33
V11	43.3	453.1341 C_28_H_22_O_6_	411, 359, 347, 317, 352, 287, 225, 185	Trans‐ε‐viniferin*	ST	17.97 ± 0.11
V12	44.2	679.1965 C_42_H_32_O_9_	571, 465, 345, 529, 501, 463, 359	Stilbene trimer	ST	22.53 ± 0.28
V13	45.5	905.1996 C_56_H_42_O_12_	811, 717, 453, 713, 475	Stilbene tetramer	ST	1.91 ± 0.04
V14	46.4	453.1340 C_28_H_22_O_6_	411, 359, 347	ω‐viniferin*	ST	3.70 ± 0.03
**Total flavan‐3‐ols derivatives (FD)**						**92.27**
**Total stilbenes (ST)**						**99.51**

Species presenting *m/z* and fragmentation pattern related to oligostilbenes [44] such as stilbene tetramers (hopaephenol, isohopeaphenol, *m/z* 905) [44, 45], stilbene trimer (*m/z* 679) and stilbene dimers such as *m/z* 471, *m/z* 453 (viniferin, trans‐ε‐viniferin, ω‐viniferin) were identified, in according to published literature [45, 46, 47, 48]. Furthermore, catechins (catechin and epicatechin) [49] were present in significant amounts and limited amount of procyanidins [49, 50] was also observed.

The chromatogram of pine bark and spruce bark extracts are reported in Figures 2 and 3, respectively. In pine and spruce bark extracts various phytoconstituents ascribable to lignans, monoterpenes, flavanols, and flavonoids were identified, as reported in Tables 3 and 4. All these compounds were previously reported for Scots pine and white spruce, as well as other varieties [45, 49, 50, 51, 52, 53].

**FIGURE 2:**
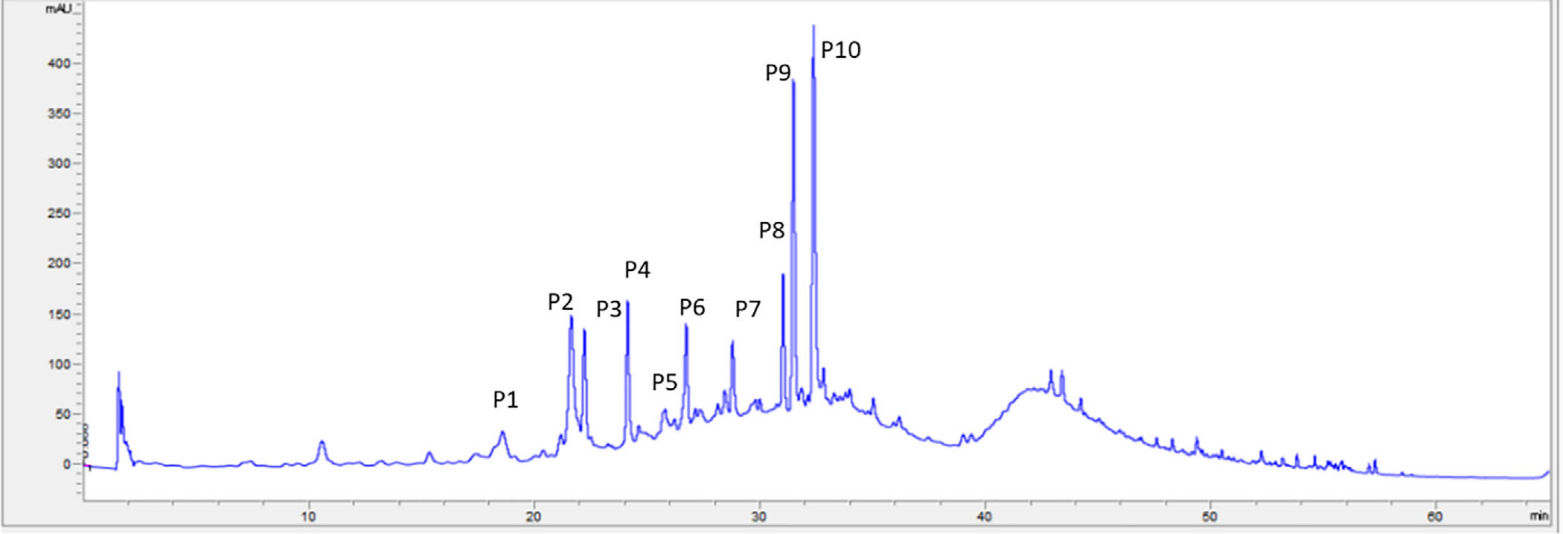
LC chromatogram of pine bark extract. Numbers are referred to the identified compounds as reported in Table 3.

**FIGURE 3:**
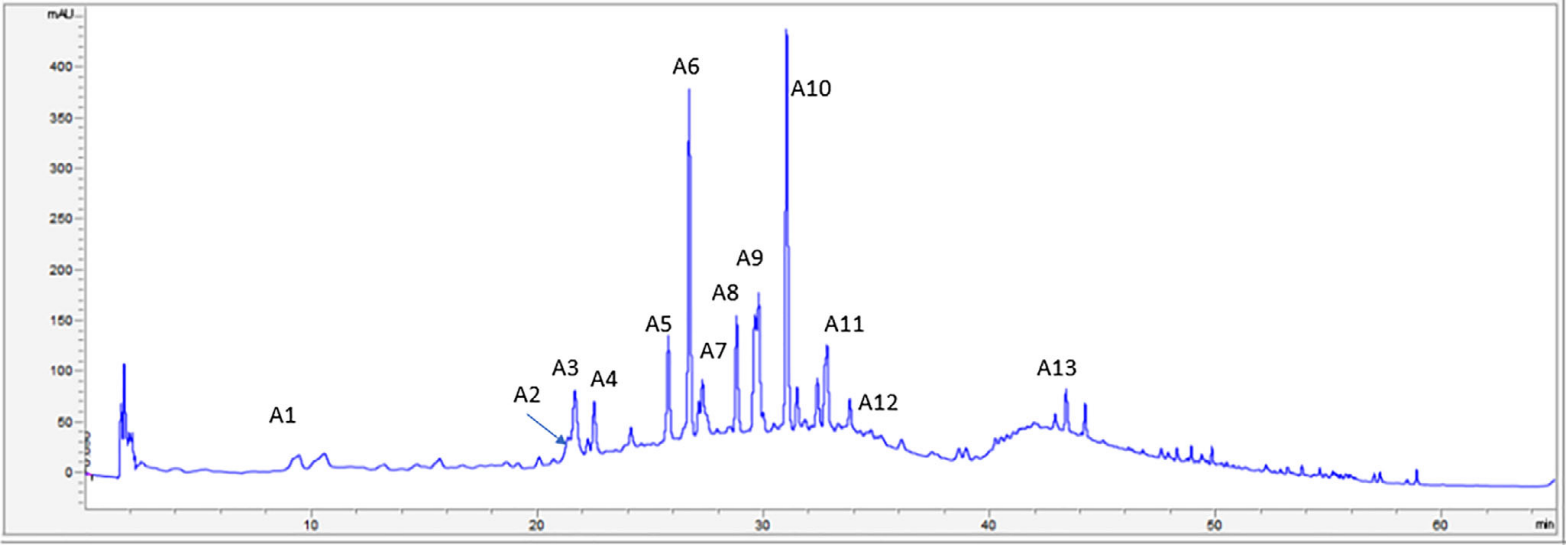
LC chromatogram of spruce bark extract. Numbers are referred to the identified compounds as reported in Table 4.

**TABLE 3:** Monomeric and oligomeric flavan‐3‐ols derivatives (FD), flavonoids (F) and sesquilignans (S) detected in pine bark extract using LC‐DAD‐MS^n^ approach. Compounds indicated with * were confirmed by reference standard injection.

N.	RT [min]	HR [M‐H]^−^ UPLC‐QTOF and molecular formula	MS/MS fragments	Annotated or Identified compounds	Chemical class	µg/mL
P1	18.4	577.1343 C_30_H_46_O_12_	425, 407, 289, 451, 287, 245, 451	Procyanidin B dimer	FD	26.63 ± 0.25
P2	21.3	289.0712 C_15_H_14_O_4_	245,205, 203, 179, 205, 227, 109, 123, 165, 125, 151	Catechin*	FD	105.18 ± 2.55
P3	22.0	589.1346 C_31_H_26_O_12_	575, 437	Methoxy oxidized procyanidin	FD	8.35 ± 0.35
P4	23.9	357.1343 C_20_H_22_O_6_	342, 327, 151, 137	Pinoresinol*	S	27.80 ± 0.98
P5	25.6	521.2052 C_26_H_34_O_11_	359 344 329 161	Lariciresinol‐O‐hexoside	S	9.05 ± 0.07
P6	26.5	553.2440 C_31_H_38_O_9_	345, 327, 315	Taxiresinol pyrocatecolglyceryl ether derivative	S	20.59 ± 1.22
P7	28.6	495.2019		Unidentified sesquilignan	S	16.54 ± 0.42
P8	30.9	491.1922 C_25_H_32_O_8_	345, 327, 315	Taxiresinol rhamnoside	S	22.55 ± 0.76
P9	31.2	465.1030 C_21_H_22_O_12_	303, 285, 241, 125	Taxifolin hexoside	F	64.79 ± 1.65
P10	32.2	303.0506 C_15_H_12_O_7_	285, 241, 125	Taxifolin*	F	85.57 ± 2.11
**Total flavan‐3‐ols derivatives (FD)**						**140.16**
**Total sesquilignans (S)**						**96.53**
**Total flavonoids (F)**						**150.36**

**TABLE 4:** Monomeric and oligomeric flavan‐3‐ols derivatives (FD), sesquilignans (S), gallic acid derivatives (GAD) and terpenes derivatives (TD) detected in spruce bark extract using LC‐DAD‐MS^n^ approach. Compounds indicated with * were confirmed by reference standard injection.

N.	RT [min]	HR [M‐H]^−^ UPLC‐QTOF and molecular formula	Formate adduct [M−H+HCOOH]^−^	MS/MS fragments	Annotated or Identified compounds	Chemical class	µg/mL
A1	10.3	305.0661 C_15_H_14_O_7_		179,219,261, 165,125	Gallocatechin *	FD	10.01 ± 0.23
A2	21.0	305.0661 C_15_H_14_O_7_		179,164,120, 221,203,175	Epigallocatechin*	FD	17.41 ± 0.88
A3	21.3	289.0712 C_15_H_14_O_4_		245,205, 203, 179, 205, 227, 109, 123, 165, 125, 151	Catechin*	FD	28.71 ± 0.01
A4	22.3	461.3529 C_21_H_18_O_12_		315,307,237, 205,419,365	Methyl ellagic acid rhamnoside	GAD	25.73 ± 0.01
A5	25.6	521.2052 C_26_H_34_O_11_		475	Lariciresinol‐O‐hexoside	S	20.34 ± 0.00
A6	26.5	553.2440 C_31_H_38_O_9_		345,315, 345,301	Taxiresinol pyrocatecol glyceryl ether derivative	S	58.77 ± 1.65
A7	27.1	475.5073 C_22_H_36_O_11_		181,293,345, 409	deoxyhexose‐hexose‐monoterpene‐ol‐one	TD	15.72 ± 0.88
A8	28.6	711.6451 C_38_H_32_O_14_			Galloylated‐flavan‐3‐ol dimer	GAD	20.13 ± 0.01
A9	29.4	525.5242 C_28_H_30_O_10_	571	345, 327, 315	Taxiresinol pyrocatecol glyceryl ether derivative	S	29.82 ± 0.11
A10	30.9	491.1922 C_25_H_32_O_8_		345, 327, 315	Taxiresinol rhamnoside	S	67.37 ± 1.15
A11	32.7	567.26395 C_25_H_44_O_14_		405, 293, 311	hexose‐hexose–hydroxy‐nor isoprenoid	TD	23.14 ± 0.22
A12	33.9	505.1342 C_24_H_26_O_12_		343	Junipegenin B 7‐O‐glucoside	F	4.28 ± 0.55
A13	42.5	519.1136 C_24_H_24_O_13_		473, 307	Isorhamnetin 3‐(2''‐acetylglucoside)	F	3.11 ± 0.08
**Total flavan‐3‐ols derivatives (FD)**							**57.11**
**Total sesquilignans (S)**							**176.30**
**Total gallic acid derivatives (GAD)**							**45.86**
**Total terpene derivatives (TD)**							**38.86**
**Total flavonoids (F)**							**7.39**

Catechin and low amount of procyanidins were detected both in spruce and pine extracts, as well as sesquilignans related to taxiresinol and lariciresinol derivatives, mainly taxiresinol pyrocatecolglyceryl ether derivatives (*m/z* 507, *m/z* 525) and two glycosides such as taxiresinol rhamnoside (*m/z* 491) and lariciresinol hexoside (*m/z* 521). In pine extract pinoresinol (*m/z* 357) as well as the flavonoids taxifolin and taxifolin hexoside was also identified, while in spruce extracts (epi)gallocatechins and other compounds derived from condensation of gallic acid such as methyl ellagic acid rhamnoside (*m/z* 461) and digalloylated dimer (*m/z* 711) as well as the glycosylated flavonoids derived from junipegenin and isorhamnetin were observed in low amount. Furthermore, terpene derivatives were detected only in spruce extract and may be derived from the aromatic fraction or essential oil of the bark.

From the quantitative point of view, sesquilignans are the most abundant compounds in spruce extract, while flavonoids were detected in pine in largest amount.

In summary the combination of the diode array spectra and multiple‐stage mass spectrometry LC‐DAD‐MS^n^ with the HR‐QTOF analysis as well as the comparison with several reference standards allowed to establish the presence of different and distinctive types of phytochemicals. The monomeric and oligomeric flavan‐3‐ols (catechin, proanthocyanidins) in all extracts, especially in branches and pine bark, oligostilbenes are characteristic of grape branches extract, the flavonoid taxifolin is peculiar of pine bark extract and sesquilignans derivatives of lariciresinol and taxiresinol were identified in pine extract and especially in spruce extract.

Based on these results and considering the presence of different classes of phytoconstituents that may have potential usefulness for the development of a cosmeceutical targeted on skin whitening and on inflammation we decided to combine the three extracts in a ratio of 1:1:1.

The three extracts were thus combined in equal parts to prepare a mixed extract to be used in the cosmetic application. The mixture (1:1:1) was also analyzed with the same LC‐DAD‐MS^n^ method and the identified compounds are reported in Table 5.

**TABLE 5:** Monomeric and oligomeric flavan‐3‐ols derivatives (FD), stilbenes (ST), flavonoids (F), sesquilignans (S), gallic acid derivatives (GAD) and terpene derivatives (TD) detected in extract mixture (1:1:1) using LC‐DAD‐MS^n^ approach. Compounds indicated with * have been confirmed by reference standard injection.

N.	RT [min]	HR [M‐H]‐ UPLC‐QTOF and molecular formula	MS/MS fragments	Annotated or Identified compounds	Chemical class	µg/mL
A1	10.3	305.0661 C_15_H_14_O_7_	179,219,261,165,125	Gallocatechin*	FD	5.23 ± 0.12
P1, V1	18.4	577.1343 C_30_H_46_O_12_	425, 407, 289, 451, 287, 245, 451	Procyanidin B dimer	FD	11.92 ± 0.05
P2, A3,V2	21.3	289.0713 C_15_H_14_O_4_	245,205, 203, 179, 205, 227, 109, 123, 165, 125, 151	Catechin*	FD	67.36 ± 0.87
A4	22.3	461.3529 C_21_H_18_O_12_	315,307,237, 205,419,365	Methyl ellagic acid rhamnoside	GAD	6.87 ± 0.88
P4	23.9	357.1343 C_20_H_22_O_6_	342, 327, 151, 137	Pinoresinol*	S	10.77 ± 0.55
P5, A5	25.6	521.2052 C_26_H_34_O_11_	359 344 329 161	Lariciresinol‐O‐hexoside	S	9.75 ± 0.05
P6, A6	26.5	553.2440 C_31_H_38_O_9_	345, 327, 315	Taxiresinol pyrocatecolglyceryl ether derivative	S	28.02 ± 0.98
A7	27.1	475.5073 C_22_H_36_O_11_	181,293,345, 409	deoxyhexose‐hexose‐monoterpene‐ ol‐one	TD	4.78 ± 0.12
P7	28.6	495.2019		Unidentified sesquilignan	S	10.42 ± 0.42
A9	29.4	525.5242 C_28_H_30_O_10_	345, 327, 315	Taxiresinol pyrocatecol glyceryl ether derivative	S	11.45 ± 0.63
A10, P8	30.9	491.1922 C_25_H_32_O_8_	345, 327, 315	Taxiresinol rhamnoside	S	29.99 ± 0.25
P9	31.2	465.1030 C_21_H_22_O_12_	303, 285, 241, 125	Taxifolin hexoside	F	23.34 ± 0.33
P10	32.2	303.0506 C_15_H_12_O_7_	285, 241, 125	Taxifolin*	F	31.95 ± 1.11
A11	32.7	567.26395 C_25_H_44_O_14_	405, 293, 311	hexose‐hexose–hydroxy‐norisoprenoid	TD	7.40 ± 0.51
V8	34.3	453.1341 C_28_H_22_O_6_		Viniferin*	ST	0.39 ± 0.01
V9	36.4	905.1996 C_56_H_42_O_12_	811, 717, 611, 357, 675, 568, 488, 359	Hopeaphenol	ST	2.43 ± 0.03
V10	38.8	905.1996 C_56_H_42_O_12_	811, 717, 611, 503, 357	Isohopeaphenol	ST	13.55 ± 0.22
A13	42.5	519.1136 C_24_H_24_O_13_	473, 307	Isorhamnetin 3‐(2''‐acetylglucoside)	F	1.09 ± 0.02
V11	43.3	453.1341 C_28_H_22_O_6_	411, 359, 347, 317, 352, 287, 225, 185	Trans‐ε‐viniferin*	ST	6.87 ± 0.25
V12	44.2	679.1965 C_42_H_32_O_9_	571, 465, 345, 529, 501, 463, 359	Stilbene trimer	ST	8.02 ± 0.14
V13	45.5	905.1996 C_56_H_42_O_12_	811, 717, 453, 713, 475	Stilbene tetramer	ST	0.65 ± 0.01
V14	46.4	453.1341 C_28_H_22_O_6_	411, 359, 347	ω‐viniferin*	ST	1.20 ± 0.05
**Total flavan‐3‐ols derivatives (FD)**						**84.51**
**Total stilbenes (ST)**						**31.91**
**Total flavonoids (F)**						**56.38**
**Total sesquilignans (S)**						**100.40**
**Total gallic acid derivatives (GAD)**						**6.87**
**Total terpene derivatives (TD)**						**12.18**

Extract mixture preserved the phytochemicals detected in single extracts, combining and adding the different chemical classes of grape branches, pine, and spruce bark. Sesquilignans were detected in extract mixture in major amounts, followed by monomeric and oligomeric flavan‐3‐ols derivatives, flavonoids, and oligostilbenes. The LC chromatogram of extract mixture 1:1:1 is reported in Figure 4.

**FIGURE 4:**
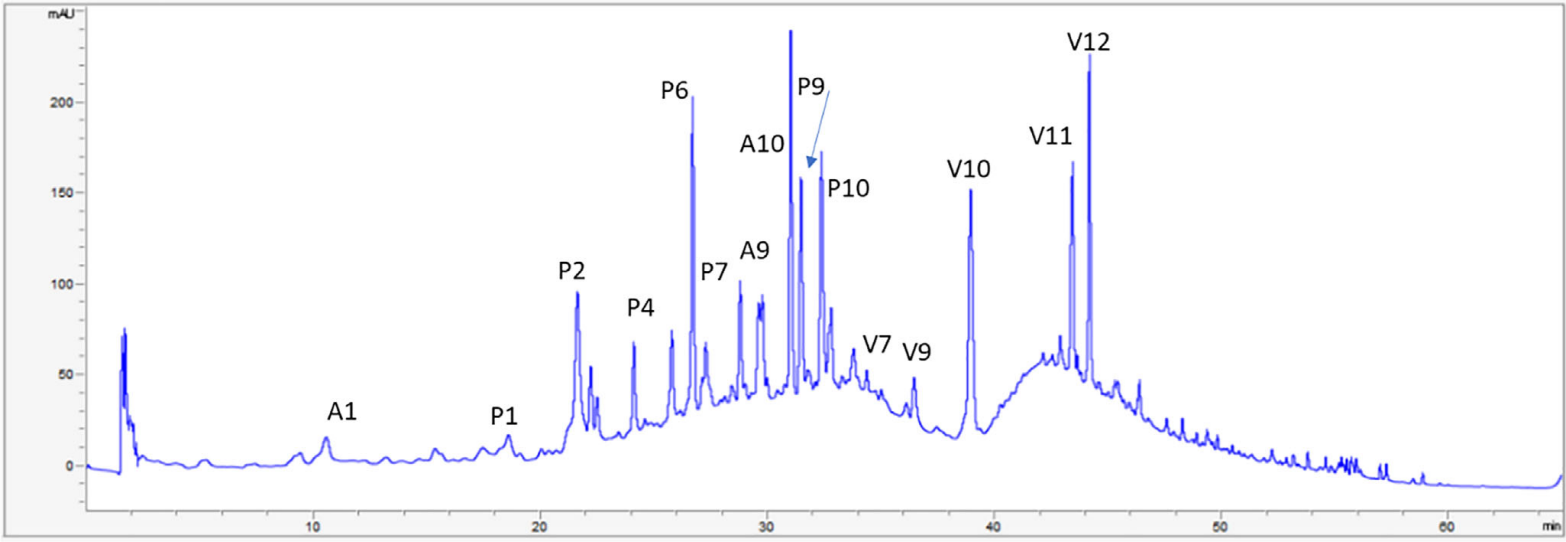
LC chromatogram of grape branches, pine and spruce bark extract mixture (1:1:1). Numbers are referred to the identified compounds as reported in Table 5.

### 4.3 Tyrosinase Inhibitory Activity Assay in Grape Branches Extract, Pine Bark Extract, Spruce Bark Extract and Extract Mixture (1:1:1)

The results related to inhibition of tyrosinase enzyme performed by grape branches, pine bark, and spruce bark extracts as well as their mixture (1:1:1) in different concentrations is reported in Figure 5.

**FIGURE 5:**
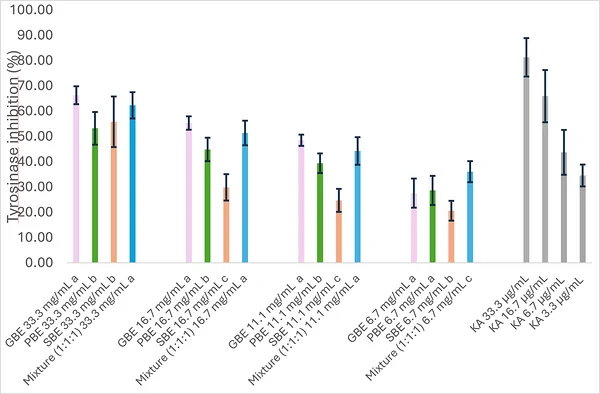
Tyrosinase inhibition % of kojic acid, grape branches extract (GBE), pine bark extract (PBE), spruce bark extract (SBE) and their mixture (1:1:1) at different concentrations. Different letters indicate statistical differences in the groups (*p* < 0.05).

These results demonstrate both for extracts, extract mixture (1:1:1) and positive control (kojic acid) an increasing tyrosinase inhibitory effect linked to the increase of tested concentration, confirming the dose‐response correlation. Considering single extracts and extract mixture, the tyrosinase inhibition ranges from 66%–53% at the highest concentration (33.3 mg/mL), decreasing to 55%–25% at medium concentrations (16.7‐11.1 mg/mL) and going to 35%–20% at the lowest concentration (6.7 mg/mL). The extract mixture preserves the tyrosinase inhibitory activity of single extracts, maintaining or improving the theorical average values (related to the equal mixture of each extract) in all concentrations tested.

### 4.4 In Vitro Evaluation of Soothing Efficacy on Reconstructed human epidermidis on Extract Mixture (1:1:1)

Data of preliminary MTT cytotoxicity assay and acceptability criteria are reported in Supporting Information and allowed to assess the compatibility of the sample with the test (24 h exposure). In preliminary cytotoxicity, propanediol was also tested to rule out adverse effects due to the solvent in which the sample is resuspended, data are summarized in Tables S1 and S2. The results of the in vitro assay are reported in Figure 6 and are expressed as pg/mL of IL‐1α released after the different treatments. The samples were as follows, PBS as negative control, SLS 0.4% as positive control, SLS 0.4 %+40 µL of extract mixtures and 40 µL of extract mixtures without SLS. The incubation of PBS on reconstructed human epidermidis produces a low amount of IL‐1α (17.1 pg/mL), as expected. Also, the incubation of the mixtures of grape branches, pine and spruce bark extracts (1:1:1) without SLS‐induced damage does not impact on the inflammatory parameter in a significant way and the value of the released IL‐1α remains quite low (45.4 pg/mL). The inflammation induced by SLS is almost 20 times higher compared to the negative control (442 pg/mL) and it is inhibited in a significant way with the addition of extract mixture (337 pg/mL). In conclusion, after a 24‐h incubation, the extract mixture produced a significant reduction (‐24%) of IL‐1α release induced by SLS damage.

**FIGURE 6:**
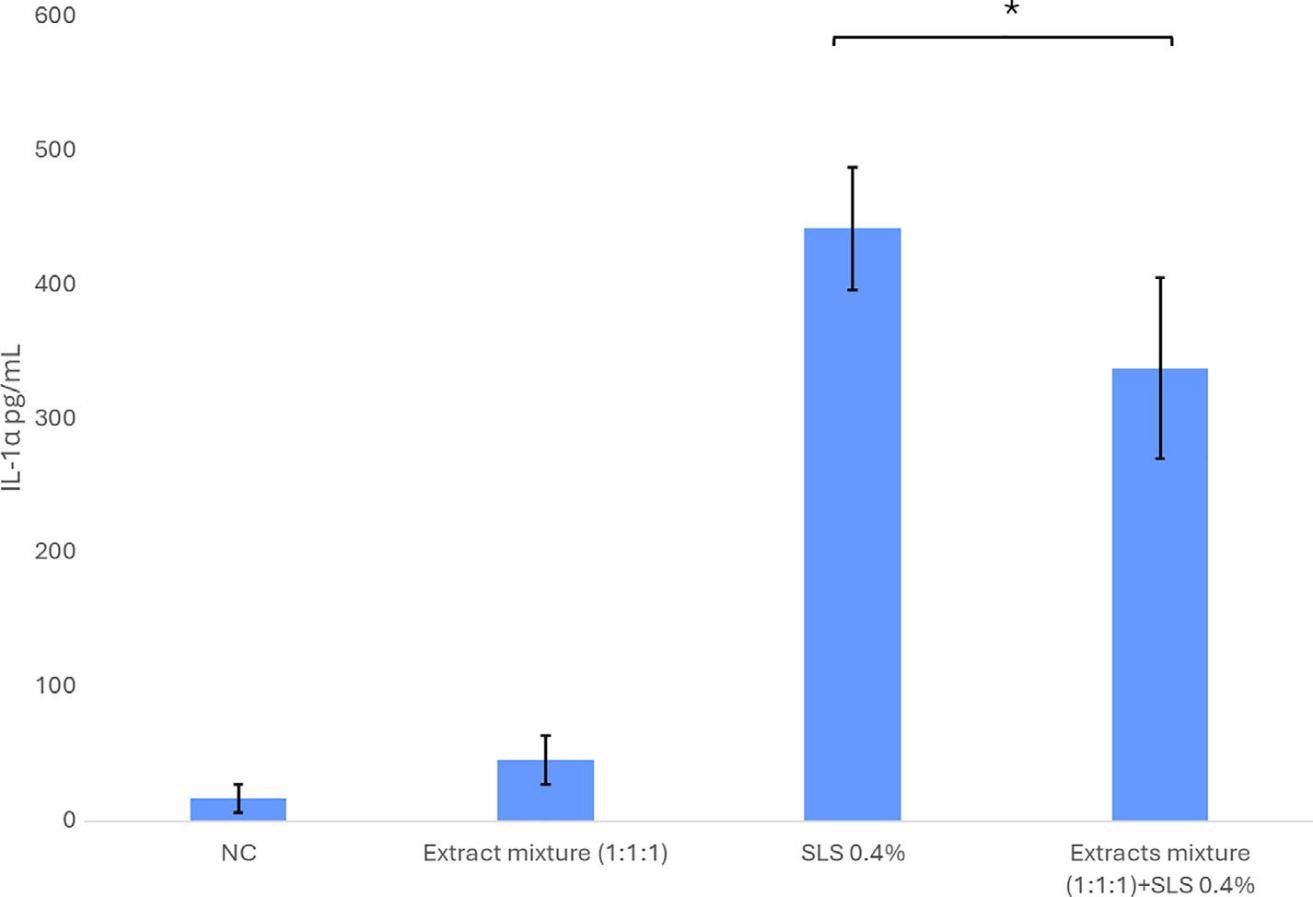
pg/mL of IL‐1α released (pg/mL) after the different treatments. (NC: negative control, SLS: sodium lauryl sulfate, *p < 0.05)

## 5 Discussion

In this work, extracts were prepared using a mixture obtained with 1,3‐propanediol:water 70:30 as extraction solvent. The extraction is obtained using plant waste materials and the process leads to the production of extracts suitable for cosmeceutical application. 1,3‐propanediol can be considered green and skin‐friendly and it is an environmentally sustainable ingredient because it is obtained by fermentation employing nonpathogenic microorganisms or from plants that produce glycerol. Indeed, referring to the technical data sheet, the 1,3‐propanediol (propanediol) employed in this study is obtained from fermentation of corn‐derived glucose and contains no added preservatives, petroleum‐based ingredients, or animal by‐products.

Among diols, propanediol presents the highest frequency of use in cosmetic applications and is employed in cosmetic products for its high humectant capacity, high solubility, solvency power [54]. Furthermore, propanediol was reported to increase the effectiveness of preservative systems and its antimicrobial effect was demonstrated to be higher compared to other glycols such as propylene glycol [55]. This antimicrobial feature together with the lower viscosity value (∼48 mPa·s) at room temperature compared to that of more conventional glycols employed in the extraction of botanicals such as glycerol (874 mPa·s) [56] make propanediol an interesting solvent for the obtainment of plant extracts for cosmetic purposes allowing both microbiological stability and easier filtration running.

The obtained extracts from grape branches, pine bark, and spruce bark were deeply investigated for their antioxidant activity by in vitro assays using different approaches (DPPH, ABTS, Phosphomolybdenum, metal chelating, CUPRAC, FRAP). The best overall antioxidant activity was detected in pine extract, suggesting a higher involvement of pine extract phytocomplex in the regulation of redox mechanisms compared to grape and spruce extracts. Furthermore, chemical fingerprinting was obtained by LC‐DAD‐MS^n^ leading to the identification and quantification of several constituents.

Flavan‐3‐ols and derivatives such as catechin and B‐type proanthocyanidins were found in grape, pine, and spruce extracts. These compounds are able to protect from the oxidative stress damage [24, 57, 58], and exert anti‐inflammatory activity through the modulation of cytokines production and prevention of excretion of eicosanoid [59]. Catechin and its derivatives, including ECG found in spruce extract, were also associated with tyrosinase inhibition and to the downregulation of its expression in melanoma cells [60]. Catechin reduces melanogenesis through the inhibition of microphthalmia‐associated transcription factor (MITF), which is involved in the development of melanocytes, thus preventing hyperpigmentation [61]. Proanthocyanidins inhibit the monophenolase and diphenolase activities of tyrosinase enzyme [57] and decrease the expression of tyrosinase and tyrosinase related protein (TRP) 1 and 2 in UV‐irradiated human melanocytes. Proanthocyanidins protect melanocytes from the effects of UV‐damage involved in melanogenesis by improving cell viability, scavenging intracellular ROS, regulating cell cycle and inhibiting protein expression of melanogenic enzymes [62].

In addition to catechin and proanthocyanidins, oligomeric (dimers‐tetramers) stilbenes derived from viniferin were found in grape branches extract. These compounds present great potential for the prevention of hyperpigmentation. ε‐viniferin, a resveratrol dimer found in grape branches extract, is reported for its antioxidant activity, through radical scavenging involving cycloaddition on the double bond of phenol moieties hydroxyl groups as proposed mechanism of action [63]. Furthermore, trans‐ε‐viniferin, was reported as a more efficient tyrosinase inhibitor compared to monomeric resveratrol and the reference compounds kojic acid and arbutin [58]. ε‐viniferin and other oligomers such as the trimer ampelopsin C and the tetramer vitisin A were studied for their anti‐inflammatory activity in comparison to resveratrol and polydatine (resveratrol glucoside) both in imiquimod (IMQ)‐activated keratinocytes and mouse skin. The in vitro inhibition of IMQ‐induced inteleukin‐1 beta (IL‐1β), interleukin‐6 (IL‐6), and interleukin‐8 (IL‐8) release was similar for all the tested stilbenoids. However, the topical application of ε‐viniferin in mouse skin with impaired barrier capacity led to a higher accumulation compared to resveratrol (0.067 vs. 0.029 nmol/mg). Furthermore, topical ε‐viniferin alleviated the inflammation induced‐symptoms and reduced IL‐23 secretion more effectively than resveratrol (58% vs. 37%), suggesting the efficiency of this compound as anti‐inflammatory agent [64].

In pine bark extract, taxifolin was detected as the most representative compound. Taxifolin is a flavonoid reported in literature for its antioxidant, anti‐inflammatory, and anti‐melanogenic activity. Taxifolin was found to be a powerful antioxidant and antiradical agent in different in vitro bioassays, when compared with reference standard antioxidants such as ascorbic acid and tocopherol, thanks to its conjugation structures and resonance stability of both phenolic rings which allow a stabilization of radicals by the subtraction of hydroxyl groups [65]. Furthermore, in Tumor necrosis factor‐alpha (TNF‐α)‐damaged keratinocytes, taxifolin significantly inhibited mRNA expression levels of pro‐inflammatory cytokines (IL‐1α, IL‐1β, IL‐6, IL‐8) and chemokines such as chemokine ligand 20 (CCL20) through the regulation of phosphorylation pathway of different kinase and transcription factors [66]. Taxifolin was reported as one of the most active flavonoids in inhibiting interferon Q (IFNQ)‐induced Intercellular Adhesion Molecule 1 (ICAM‐1) protein in human keratinocytes, with a critical role in the regulation of cutaneous inflammation, immunologic reactions and skin repair [67]. Taxifolin was also found to inhibit tyrosinase‐catalyzed oxidation of l‐dihydroxyphenylalanin, cell pigmentation induced by expression of exogenous human tyrosinase [68] and collagenase‐1 in UVB irradiation assay [68].

Sesquilignans, especially taxiresinol and lariciresinol derivatives, are the main compound classes detected in spruce bark extract as well as pinoresinol represents the major sesquilignan in pine bark extract. Lignans are highly distributed in plants and coniferous tree especially Norway or red spruce [53] and they are described in literature for their antimicrobial, antioxidant, and anti‐inflammatory activities. Flaxseed lignans, including lariciresinol, were already proposed as possible cosmetic active ingredients for anti‐age formulations [69]. The inclusion of flaxseed lignans in cosmetics prevents skin sagging, contributes to the maintenance of skin elasticity, with the aim to treat dry, rough, and lackluster skin [70]. Furthermore, lignans are also reported as natural stabilizers since their inclusion prevents the oxidation of oily ingredients in emulsions, extending the shelf lives of the formulations [70]. Taxiresinol was reported as the most promising lignan in the inhibition of histamine release from the human basophilic cell line [71]. Histamine plays important roles in inflammation and nervous irritability in allergic disorders, including atopic dermatitis [72]. Furthermore, even lignan‐derived compounds such as pinoresinol were studied in vitro for their tyrosinase inhibitory activity, taxiresinol, lariciresinol as well as lignans in general are less investigated compared to flavan‐3‐ols derivatives, stilbenoids, and flavonoids, so this study may represent a pioneering investigation of taxiresinol and lariciresinol bioactivity for cosmetic applications targeting melanogenesis [73].

The phytochemical investigation of grape branches, pine bark, and spruce bark extracts revealed a pool of different compounds (catechins, procyanidins, stilbenoids, flavonoids, and lignans) all previously studied as possible cosmetic actives. Previously described literature reports antioxidant, anti‐inflammatory, and anti‐tyrosinase as well as anti‐age effects suggesting the potential of grape branches, pine, and spruce bark extracts as preventive anti‐melanogenesis active ingredients for cosmetic formulation counteracting oxidation, inflammation, and melanin production.

Considering the above results and evaluating the potential usefulness of all the ingredients, a mixture of grape branches extract, pine bark extract, and spruce bark extract in equal proportion (1:1:1) was prepared, to combine all the active compounds in a single ingredient characterized from a high structural molecular diversity. LC‐DAD‐MS^n^ and UPLC‐QTOF analysis of extract mixture revealed a comprehensive presence of all chemical classes identified in each extract, suggesting a rich profile of natural substances derived from the different sources and the potentiality of further in‐depth evaluation, such as inhibition of tyrosinase activity.

The anti‐tyrosinase efficacy was evaluated both in single extracts and extract mixture (1:1:1) at different concentrations, confirming a dose‐related activity in all tested samples as well as the efficacy of the combined extract mixture compared to single extracts. These preliminary positive results suggest the appeal and the suitability of extract mixture intended as unique combination of active ingredients for the formulation of cosmetic and cosmeceutical products with whitening purpose.

For this reason, in addition to the previous preliminary assays which confirmed antioxidant effects in single extracts and the anti‐tyrosinase activity both in single extracts as well as their combination, the overall anti‐inflammatory activity of the extract mixture (1:1:1) was assessed in human reconstructed epidermidis as a more complex in vitro experimental model. IL‐1 was chosen for the assay because it is a highly active pro‐inflammatory cytokine and mutations or alterations involving its maturation or IL‐1 receptor antagonist may produce useless inflammatory conditions, especially in skin. Among cells, keratinocytes produce high levels of IL‐1, especially IL‐1α which induces an inflammatory response in the skin [74]. In particular, IL‐1α released by UVB exposure stimulates the production of keratinocyte growth factor (KGF) in keratinocytes, which promotes in turn the expression of tyrosinase. The combination between IL‐1α and KGF promotes melanogenesis through supporting melanin deposition [51]. The extract mixture (1:1:1) significantly inhibits the release (‐24%) of IL‐1α, suggesting its efficacy in preventing inflammation opening to melanogenesis and confirming soothing effect toward epidermidis.

Therefore, this study valorizes waste plant materials by the development of upcycled grape branches, pine bark, and spruce bark extracts using skin and environmentally friendly solvents (1,3‐propanediol and water) and highlights different mechanisms of action involving the overall phytoconstituents of extracts and their mixture relating to the counteract of oxidation, tyrosinase enzyme, and inflammation linked to melanin production.

## 6 Conclusion

This work evaluated the chemical composition and efficacy for cosmeceutical application of grape branches, pine and spruce barks‐hydro propanediol extracts derived from waste of local farm and sawmills. Chemical analysis yields a detailed fingerprint of the three extracts revealing the presence of several bioactive constituents such as oligomeric proanthocyanidins, stilbenoids (grape branches), the flavonoid taxifolin (pine bark), and sesquilignans (spruce bark) as significant compounds.

All these compounds are reported in literature for their antioxidant and anti‐tyrosinase activity and related in vitro spectrophotometric assays were performed as preliminary screening, confirming the efficacy of single extracts for these targets.

Furthermore, grape branches, pine, and spruce bark extracts were assembled in a single mixture (1:1:1) with the aim of combining all the detected phytochemicals in a single ingredient and to verify the efficacy of the obtained new ingredient. The mixture confirms tyrosinase inhibitory activity, and it was also tested in vitro for its anti‐inflammatory efficacy in human reconstructed epidermidis, confirming an inhibition (‐24%) of melanogenesis‐involved cytokine IL‐1α.

The results of the present work show that grape branches, as well as pine and spruce bark, along with their combination, might serve as promising active ingredients for cosmeceutical use. These extracts valorize waste sources and offer a wide range of active compounds, paving the way for future research and experiments aimed at preventing melanogenesis and various inflammatory skin conditions.

## Author Contributions

**Marta Faggian**: validation, investigation, formal analysis, visualization. **Silvia‐Maria Stanic**: investigation, visualization, data curation. **Giulia Galizia**: writing – original draft, validation. **Sara Ferrari**: investigation, supervision, data curation. **Gabriele De Nadai**: data curation, validation, visualization, methodology. **Stefano Francescato**: validation, resources, visualization. **Giovanni Baratto**: validation, resources, visualization. **Gökhan Zengin**: investigation, conceptualization, validation, methodology, formal analysis, writing – review and editing, writing – original draft, supervision, data curation. **Alessandra Semenzato**: validation, resources, visualization. **Alina Yerkassymova**: formal analysis, investigation, data curation. **Stefania Sut**: investigation, formal analysis, data curation. **Stefano Dall'acqua**: conceptualization, investigation, formal analysis, project administration, data curation, writing – review and editing, writing – original draft, methodology.

## Conflicts of Interest

Conflicts of Interest Disclosure To the Editorial Board of Chemistry and Biodiversity I am writing as the corresponding author, on behalf of all coauthors, regarding our abovementioned manuscript. The paper describe a research performed in cooperation by two Academic groups, one in Padova University one in Konya University, and two companies one is a Spin off company of Padova University (Uni R&D) and one is a Italian company focused on the production and commercialization of cosmetics and food supplements (Unifarco spa). Authors are from all the institutions. In accordance with industry ethical standards and journal's guidelines, we wish to formally disclose a potential financial conflicts of interest relating to the funding of this study. This research project was supported by funding from Unifarco spa. To ensure complete transparency and maintain scientific integrity, we declare that: Â· Authors, Sara Ferrari, Gabriele De Nadai, Gianni Baratto, Stefano Francescato are UNifarco employee. Authors Marta Faggian, Giulia Galizia, Silvia Stanic are employee of Unired Spin off. Alessandra Semenzato and Stefano Dallâ€Acqua are Academic at Padova University and partecipants at Unired Spin off. The funding company (Unifarco) provided financial support for purchasing materials reagents and some of the test but had no role in the study design. The collection, analysis, interpretation of the data, writing of this manuscript were operated by the authors and discussed in team. The decision to submit this paper for publication was made entirely by the authors, independent of any funding company approval or veto power.

## Supporting information




**Supporting File**: cbdv71916‐sup‐0001‐SuppMat.docx.

## Data Availability

The data that support the findings of this study are available from the corresponding author upon reasonable request.
