# Supplementary material for: Cosmeceutical Ingredients From Plant Waste: Bioactive Extracts From Vitis vinifera L. branches, Pinus sylvestris L. and Abies alba Mill. Barks
Source: Chem Biodivers. 2026 Nov 14;23(11):e71916. doi: 10.1002/cbdv.71916 (PMC13769031; doi:10.1002/cbdv.71916)
Supplement: Supplementary file 1 [file CBDV-23-e71916-s001.docx]

**SUPPLEMENTARY MATERIAL**

1. ***In vitro* MTT cytotoxicity evaluation on the skin cells.**

The preliminary MTT cytotoxicity assay was performed treating the reconstructed human epidermidis with the following substances: Phospate buffer (PBS) as negative control; 0.4% sodium lauryl sulphate (SLS) as positive control; 1,3-propanediol to test the component of extraction vehicle, that was composed of 1,3-propanediol; water 70:30); extract mixture (1:1:1); extract mixture (1:1:1)+0.4% SLS.

The preliminary MTT cytotoxicity assay confirms the compatibility of all tested samples with the selected experimental model and the results are reported in table 1. The cell viability after 24 hours of exposure was 100±0.7% for PBS (negative control), 74.1±0.5% for 1,3 -propanediol and 85.9±6.4% for extract mixture (1:1:1) alone, confirming good viability of these tested samples. As highlighted by the data in table 1, the MTT results of 0.4% SLS alone and extract mixture (1:1:1)+0.4% SLS confirm the impact of SLS as stressful agent on cell viability.

As shown in figure 6 of the manuscript, the inhibition of IL-1α production is statistically higher for extract mixture (1:1:1)+0.4% SLS compared to 0.4% SLS alone. These results are not affected by cell viability, because the cell mortality of mixture (1:1:1)+0.4% SLS versus 0.4% SLS alone is comparable (97-98%) and does not impact on IL-1α production.

| **Sample** | **% mean cell viability** | **% cell mortality** |
| --- | --- | --- |
| Negative control (PBS) | 100±11.7 | - |
| Positive control (SLS 0.4%) | 1.6±0.4 | 98.4 |
| 1,3 -Propanediol | 74.1±0.5 | 25.9 |
| Extract mixture 1:1:1 | 85.9±6.4 | 14.1 |
| Extract mixture 1:1:1 + SLS 0.4% | 3.0±0.1 | 97 |

Table S1: MTT preliminary cytotoxicity assay

**Acceptability criteria**

Considering the cytotoxic activity the table S2 indicate the acceptability criteria

**Ta**

**Table S2 acceptability criteria.**
